# Analysis of Burnout Syndrome and Resilience in Nurses throughout the COVID-19 Pandemic: A Cross-Sectional Study

**DOI:** 10.3390/ijerph181910470

**Published:** 2021-10-05

**Authors:** Noel Rivas, María López, María-José Castro, Sofía Luis-Vian, Mercedes Fernández-Castro, María-José Cao, Sara García, Veronica Velasco-Gonzalez, José-María Jiménez

**Affiliations:** 1 Hospital Clínico Universitario Valladolid, 47003 Valladolid, Spain; nrivas@saludcastillayleon.es (N.R.); mefernandezc@saludcastillayleon.es (M.F.-C.); 2 Nursing Faculty, University of Valladolid, 47005 Valladolid, Spain; mariajose.castro@uva.es (M.-J.C.); mjcao@enf.uva.es (M.-J.C.); sara.garcia.villanueva@uva.es (S.G.); veronica.velasco.gonzalez@uva.es (V.V.-G.); josejimenez@enf.uva.es (J.-M.J.); 3 CEIP Antonio Machado, 47011 Valladolid, Spain; sofia.luisvian@hotmail.com

**Keywords:** COVID-19, burnout, professional, nurses, resilience, psychological

## Abstract

Background: The COVID-19 pandemic has contributed to the occurrence of work-related stress on nursing staff. Being resilience an essential element to countering adversity. The aim of the study was to assess burnout syndrome as well as resilience in hospital-care nurses during the first outbreak of the COVID-19 pandemic. Methods: A cross-sectional descriptive study of burnout syndrome and resilience of 101 nurses during the first COVID-19 outbreak. The Maslach Burnout Inventory and the Scale of Resilience of Connor-Davidson were used. Results: The burnout average score was 74.35 ± 12.78 points, and resilience was 27.94 ± 5.84. Temporary nurses reached a lower average score for the emotional fatigue dimension (23.80 ± 10.39 points) *p* < 0.05. The emotional fatigue dimension correlated adversely with the average score of resilience (r = −0.271; *p* < 0.001). Conclusions: The level of burnout in nurses was high, being higher on those who took care of COVID-19 patients. Resilient nurses were able to better cope with stressful situations.

## 1. Introduction

The COVID-19 pandemic has placed all countries under extreme medical and economical strain. The 7818 cases registered around the world until 30 January 2020 caused the World Health Organization (WHO) to begin taking measures, establishing the highest health alert in response to inaction and declaring the COVID-19 outbreak as a pandemic on 11 March 2020 [1].

Cumulative cases worldwide between 31 December 2019 and 2 November 2020 were 4,659,7299 (1,201,162 deaths), with 10,324,515 in Europe with Spain being the third European country with the greater impact at that time (1,185,678 cases) [2].

In Spain, according to Red Nacional de Vigilancia Epidemiológica (RENAVE) the largest amount of cases occurred within the 15–65-year group (68% of total). [3].

The COVID-19 pandemic has increased the demand for nurses needed to provide quality healthcare; it has likewise showed the need to guarantee a strong and enduring workforce. Workload, temporary staff recruitment, reduction of autonomy, and continuous organizational change are a hardship for the nursing workforce. This situation, along with the pressure of working pressure and the insecurity forces nurses to deal with high stress levels and anxiety [4]. Burnout is a challenges with which nurses are frequently confronted; it linked to their job, as are stress levels and responsibility [5]. Maslach defines burnout as a set of symptoms involving a physical and psychological dimension, including negative attitudes derived from work and personal relationships that lead to exhaustion, fatigue, distress, and despair [6].

Continuous exposure to stress situations may have negative impact on mental and physical health; therefore, there could be a significant impact on job performance. The COVID-19 pandemic could result in high levels of anxiety in nurses with major stress loads [7].

Adversity in light of the increase in workload along with the lack of material resources were described beforehand as factors that raised stress amongst nursing staff in a crisis; being resilience is the optimal way to deal with change effectively [8]. Nurses can be overwhelmed by stress, affecting their physical and mental well-being [9]. Resilience is defined as a person’s multidimensional learning capacity that enables them to function at a high level when dealing with adversity [10]. Resilience contributes to reducing the negative impact of stress within the working environment, as well as to adapting to work challenges and improving personal resources [11]. Resilience can be a predictor of burnout in nurses, due to its association with exhaustion and its influence on it [12]. Higher levels of personal resilience and social and organizational support have been associated with reduced levels of anxiety related to the COVID-19 pandemic [7].

In the workplace, specific interventions that foster the well-being of nurses should be promoted [13]. These are necessary to address organizational barriers and the risks of burnout and stress [14]. Yöruk et al. consider that psychological difficulties may cause other permanent long-term problems if they are not treated in time. It is important to protect the mental well-being as well as the physical health of health professionals facing COVID-19. Resilience is crucial to adapt and strive against labor challenges and to reduce symptoms of stress, emotional burnout, cynicism, and depression [15]. 

The work pressures suffered by nurses in an overloaded and under-resourced healthcare system have an impact on their mental state [16]. Therefore, those responsible for the management of healthcare personnel should plan emotional support and physical, psychological, and reciprocal social strategies [4]. The pandemic caused by COVID-19 has shown a lack of coordination of material and human resources in Spain as in other healthcare systems [17]. To identify the impact of health in health personnel will allow us to address strategic actions to reduce burnout and to foster resilience [18].

The present study proposes to assess burnout syndrome and resilience of nurses during the COVID-19 pandemic to establish future actions which will allow us to improve situations and to deal with work stress.

## 2. Materials and Methods

### 2.1. Study Design

This was a descriptive cross-sectional study of burnout syndrome and resilience in registered nurses during the first outbreak of the COVID-19 pandemic.

### 2.2. Sample and Study Participants

The study was carried out in a third-level hospital of the Regional Health Service in Spain, on a population of 1022 active nurses in hospitalization units and special services from 1 March to 30 May 2020.

The sample was made up of 101 nurses who answered voluntarily and anonymously the following questionnaires: the Maslach Burnout Inventory and the Scale of Resilience of Connor-Davidson (10-item CD-Risc) [6,19]. These questionnaires were answered during May 2020 through the hospital’s intranet system. Nurses who were on sick leave during the period of the study were excluded.

### 2.3. Data Collection and Variables of the Study

During May 2020, digital access to a questionnaire was provided through the hospital’s intranet system. In order to fill in the questionnaire, it was necessary to read the purpose of the study as well as to explicitly agree to participate. The form consisted of the Maslach Burnout Inventory questionnaire and the Scale of Resilience of Connor-Davidson (10-item CD-Risc). The following socio-demographic variables were used: age, gender, working unit, and type of employment contract.

In order to analyze burnout syndrome, a validated tool scale of the Maslach Burnout Inventory questionnaire was used in its Spanish-adapted version [20] which is composed of 22 items with a score of 0 to 6 points: 0 = never, 6 = on a daily basis. (Table S1: Maslach Burnout Inventory questionnaire scale, version adapted to Spanish). The questionnaire is divided into three dimensions:

- Emotional fatigue or emotional burnout. Questions 1, 2, 3, 6, 8, 13, 14, 16, and 20.
- Cynicism. Questions 5, 10, 11, 15, and 22.
- Personal fulfilment. Questions 4, 7, 9, 12, 17, 18, 19, and 21.

Answers were categorized according to the average score obtained and the following classification:

- Emotional fatigue or emotional burnout. Low < 15 points, medium 15–24 points, high > 24 points.
- Cynicism. Low < 4 points, medium 4–9 points, high > 9 points.
- Personal fulfilment. Low < 33 points, medium 33–39 points, high > 39 points.

The reliability of the questionnaire as per α of Cronbach ranged from 0.72 to 0.90 [19]. To analyze resilience, the Scale of Resilience of Connor-Davidson (10-item CD-Risc) validated in its Spanish version was also used [20]. This is composed of 10 items measured through the 0–4 point Likert scale (0 = totally disagree, 4 = totally agree). (Table S2: Connor-Davidson Resilience Scale version adapted to Spanish).

In the validation of the Spanish version, a Cronbach’s α reliability of 0.85 was obtained [21]. 

### 2.4. Ethical Considerations

Participation was voluntary and data processing was anonymous and confidential in accordance with current regulations. The research followed the rules on bioethics established in the Declaration of Helsinki and its latest update. This project was approved by the Clinical Research Ethics Committee (reference number: PI 20-1831).

### 2.5. Statistical Analysis

Data were analyzed with the IBM SPSS v. 22.0 software (IBM, Armonk, New York, NY, USA). A descriptive analysis was carried out through centralization measures for quantitative variables and through frequencies for qualitative variables. To establish the reliability of the scales, an analysis of the internal validity was undertaken based on the determination of the coefficient α of Cronbach. A Chi-square test was used to assess the association between the sociodemographic variables and the results of the survey for qualitative variables and analysis of variance (ANOVA). A *p* value < 0.05 was considered statistically significant.

## 3. Results

A total of 101 registered nurses participated in the study with an average age of 41.27 ± 10.03 years old; 93 were women (41.27 ± 10.11 years) and 8 were men (41.25 ± 9.72 years).

With regards to the assistance unit, 54 nurses (53.46%) developed their work in units aimed exclusively at patients with COVID-19 whereas 47 nurses (46.54%) developed theirs in units aimed at patients with COVID-19-free pathologies.

During the course of the pandemic, 31 nurses (30.69%) were switched from their assistance position to another hospital unit by the nursing management. The average age of the shifted nurses was lower than that of who maintained their usual job position (38.58 ± 9.97 years vs. 42.46 ± 9.89 years). 

The type of employment contract of the nurses who participated in the study was mainly a non-permanent contract and their average age was lower than the average age of the permanent staff (Table 1).

The average score of the Maslach Burnout Inventory scale in the Spanish version was 74.35 ± 12.78 points, without showing significant differences between men (72.65 ± 14.59 points) and women (74.50 ± 12.69 points), *p* = 0.69. 

‘Emotional fatigue or emotional burnout’ dimension showed an average score of 29.53 ± 11.03 points, the ‘cynicism’ dimension average score was 9.20 ± 5.95 points, and ‘personal fulfilment’ dimension average score was 35.62 ± 6.55 points. With respect to gender, the ‘emotional fatigue or emotional burnout’ dimension revealed an average score in women of 29.61 ± 11 points and in men of 28.62 ± 12.16 points (*p* = 0.809). In the ‘cynicism’ dimension, the average score for women was 9.05 ± 5.96 points and for men 10.87 ± 5.93 points (*p* = 0.409). Lastly, in the ‘personal fulfilment’ dimension the average score for women was 35.83 ± 6.61 points and 33.12 ± 5.43 points for men (*p* = 0.263).

The average scores of the Maslach Burnout Inventory scale obtained in the Spanish version were analyzed in relation to the type on employment contract, showing statistically significant differences (Table 2).

Nurses who worked in units with patients with COVID-19 showed a higher average score (76.5 ± 11.89 points vs. 71.89 ± 13.44 points; *p* = 0.071) as well as in ‘emotional fatigue or emotional burnout’ (32.24 ± 11.14 points vs. 26.42 ± 10.14 points; *p* = 0.008) and ‘cynicism’ dimensions (9.51 ± 5.78 points vs. 8.82 ± 6.18 points; *p* = 0.56). However, the ‘personal fulfilment’ dimension average score was higher in nurses who worked in units with patients with non-COVID-19 pathologies (36.73 ± 4.78 points vs. 34.74 ± 7.70 points; *p* = 0.135).

**Figure 1:**
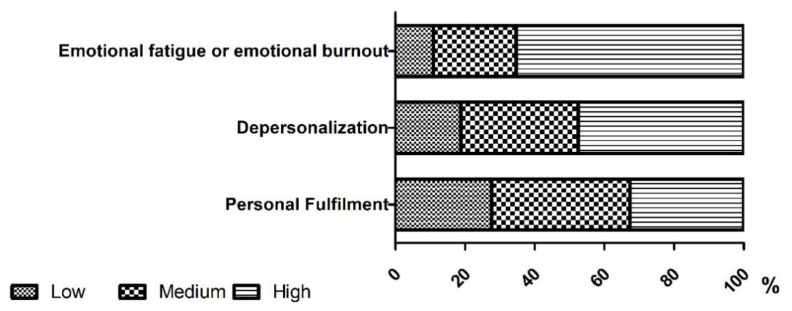
Categorization of burnout risk in each of its dimensions.

Figure 1 illustrates the distribution of the results obtained when categorizing the three dimensions in the burnout evaluation questionnaire. A high inclination was observed in the ‘emotional fatigue or emotional burnout’ and ‘cynicism’ dimensions. However, there was a lower risk in the ‘personal fulfilment’ dimension. (Figure 1).

‘Emotional fatigue or emotional burnout’ remained high in nurses who worked in units with patients with COVID-19, compared to those nurses who worked in units with patients with non-COVID-19 pathologies (n = 41.75.9% vs. n = 25.53.2%; *p* < 0.05).

With respect to resilience analysis, the average score was 27.94 ± 5.84 points, without observing significant differences by gender. Women had an average of 28.07 ± 5.93 points compared to the 26.37 ± 4.65 points of men; *p* = 0.432. Likewise, major differences were not observed in relation to the average results obtained between nurses in units with patients with COVID-19 (28.46 ± 5.91 points) and nurses in units with patients with non-COVID-19 pathologies (27.34 ± 5.76 points), *p* = 0.338. 

When studying the results obtained according to the type of employment contract, significant differences were not observed in the resilience average score, even though nurses with a temporary contract showed a higher score (30.28 ± 4.32 points) than interim nurses (26.60 ± 6.69 points) and permanent nurses (28.05 ± 5.31 points), *p* = 0.062.

The total average score of burnout did not relate significantly to the average score of resilience. The ‘Emotional fatigue or emotional burnout’ dimension related adversely to the average score of resilience (r = −0.271; *p* < 0.001). The ‘cynicism’ dimension showed a negative correlation without being statistically significant. The ‘personal fulfilment’ dimension showed a positive correlation with the average score of resilience (r = 0.288; *p* < 0.001).

## 4. Discussion

The first outbreak of the COVID-19 pandemic caused high working pressure as well as the need for quick decision-making with ethical implications, which made nurses vulnerable to stress [22]. This is reflected in the results of our study which reveal evidence of burnout within the nurses who participated in it.

The level of burnout in the nurses of the study was high. The ‘emotional fatigue or emotional burnout’ dimension (average score: 29.53 ± 11.03 points) was comparable to other studies (29.13 ± 10.30 points and 32.21 ± 12.01 points, respectively [23,24]). The results of the study regarding the ‘cynicism’ dimension (9.20 ± 5.95 points) were more favorable than scores in other studies [23,24]. Discrepancies in the results of the ‘personal fulfilment’ dimension in the aforementioned studies were observed; the study by Jose et al. showed a better score (37.68 ± 5.17 points) in contrast to our result (35.62 ± 6.55 points). 

In our study, the nurses with the highest levels of stress were those with more job stability—permanent and interim nurses compared to temporary nurses. This may be due to the fact that the increase in recruitment of nurses during the pandemic meant that newly qualified nurses entered the labour market without having experienced previous work overload and with a high level of motivation. Regardless of career prospects, this group was also the most resilient.

Other investigations that analyze heterogeneous samples of nurses who work in different departments have shown that age, sex, or length of service are not significantly associated with presenting stress levels [25]. 

Nurses who took care of patients with COVID-19 showed a higher level of burnout than those nurses who took care of patients with non-COVID-19 pathologies. This was especially evident in the higher average score in the ‘emotional fatigue or emotional burnout’ dimension. Other projects also reflect the relationship between high levels of stress and tiredness and caring for patients with COVID-19 [26,27,28]. In contrast, nurses who took care of patients with other pathologies showed a better score in the ‘personal fulfilment’ dimension. Working with COVID-19 patients is considered to be a health threat and leads to exhaustion, also linked to psychosocial factors that claim healthcare resources. These are all reasons for the appearance of burnout amongst nurses [29]. Continued work activity during the pandemic may have been a risk factor in the occurrence of burnout, mainly among nurses who had to attend COVID-19 patients. The implementation of exceptional safety measures in patient management, together with the initial lack of knowledge and the lack of effective management of the pandemic in the health care setting, may have been another factor that justified a greater predisposition to emotional exhaustion among nurses.

In our study, temporary contracts predominated in younger nurses who obtained a lower average score in the ‘emotional fatigue or emotional burnout’ dimension, in comparison with nurses with an interim or permanent contract. Regarding the ‘personal fulfilment’ dimension, nurses with a temporary contract showed the highest score followed by nurses with a permanent contract and interim nurses. These results contrast with the results obtained in other studies, which show that young nurses suffer a higher level of burnout than older and more experienced nurses due to a lack of working experience [28].

Our study observed a strong connection between ‘emotional fatigue or emotional burnout’ and resilience, in the same way as the relationship between ‘personal fulfilment’ and resilience. Similar results are gathered in studies such as that by Jose et al. that showed an increase of resilience within nurses, which helped to mitigate burnout signs [23], allowing a positive adaptation to situations with a high workload and work-related stress.

Resilience is necessary to cope with the health crisis caused by COVID-19 and arises as the moderating variable able to reduce the relationship between the negative effect and compassion satisfaction [25]. Resilience, as a single feature based on traits, becomes a coping mechanism to adversity in each person so it is necessary to foster it [30].

The health and social situation in which nurses are involved during the COVID-19 pandemic creates a high working pressure and can also trigger psychological issues [21]. In order to continue with their job, nurses will need moral support and a strong leadership that designs strategies to reduce the stress to which they are subjected [31]. Resilience should also be promoted bearing in mind the current social context which can be a barrier to the development of a positive adaptation [32].

Implementing support strategies within health institutions would allow them to improve the work-related stress management of their employees. Facing a health crisis like to one caused by COVID-19, an appropriate human and resources management is essential in order to prevent and cope with work-related stress and to handle related consequences. The type of contract of nurses in healthcare institutions could be a predictor of burnout syndrome [12].

Within the constraints of this study it is worth highlighting the lack of randomness of the analyzed sample as well as the sample selection in which nurses of hospitalization and special units participated voluntarily. It would be necessary to repeat the same study not only at a hospital-care level, but also in primary and home care. Nevertheless, these aspects did not alter the main aim of the study. 

## 5. Conclusions

This work shows the stress situation nurses must deal with in a health crisis. The analysis of burnout syndrome demonstrates a prevalence of ‘emotional fatigue or emotional burnout’ in nurses during the first outbreak of COVID-19. Nurses who took care of patients with COVID-19 featured a higher level of burnout. 

Nurses with higher average scores in resilience better faced up to emotional burnout and cynicism.

The type of working contract may be a predictor of the level of stress and resilience. In order to achieve an effective management system that helps to improve positive attitudes and human relations, nursing administrations should consider this in order to plan strategies to reduce burnout and increase resilience.

## Figures and Tables

**Table 1:** Working situation and age of nurses.

	Type of Employment Contract			*p*-Value
	Temporary N (%) 21 (20.79%)	Interim N (%) 40 (39.6%)	Permanent N (%) 40 (39.6%)	
Average age (years)	30.38 ± 7.45	39.33 ± 5.81	48.93 ± 8.24	<0.001

**Table 2:** Average score of burnout and dimensions as per type of employment contract.

		Type of Employment Contract			*p*-Value
		Temporary N (%) 21 (20.79%)	Interim N (%) 40 (39.6%)	Permanent N (%) 40 (39.6%)	
Dimension	Emotional fatigue or emotional burnout	23.80 ± 10.39	30.67 ± 8.76	31.40 ± 12.54	0.025
	Cynicism	9.95 ± 5.84	10.07 ± 6.49	7.92 ± 5.33	0.221
	Personal Fulfilment	38.38 ± 5.73	33.90 ± 7.11	35.90 ± 5.93	0.036
Total Burnout		72.14 ± 11.63	74.65 ± 11.89	75.22 ± 14.30	0.663

## Supplementary Materials

The following are available online at https://www.mdpi.com/article/10.3390/ijerph181910470/s1, Table S1: Maslach Burnout Inventory questionnaire scale, version adapted to Spanish, Table S2: Connor-Davidson Resilience Scale version adapted to Spanish.
