# Supplementary material for: Analysis of Burnout Syndrome and Resilience in Nurses throughout the COVID-19 Pandemic: A Cross-Sectional Study
Source: Int J Environ Res Public Health. 2021 Oct 5;18(19):10470. doi: 10.3390/ijerph181910470 (PMC8508575; doi:10.3390/ijerph181910470)
Supplement: Supplementary file 1 [file ijerph-18-10470-s001.zip › ijerph-1384727-supplementary.pdf]

Table S1 — Maslach Burnout Inventory questionnaire scale, version adapted to Spanish

Likert-type answer with a score of 0-6.

0= Never.

1= A few times a year or less.

2= Once a month or less.

3= A few times a month.

4= Once a week.

5= A few times a week.

6= Every day.

1. I feel emotionally exhausted by my job.
2. I feel tired at the end of a working day.
3. When I get up in the morning and I face another working day I feel exhausted.
4. I easily understand how my patients feel.
5. I believe I am treating some patients as if they were impersonal objects.
6. I feel that working during the whole day with a lot of people is a great effort and it is tiring.
7. I think I handle my patients' problems very effectively.
8. I feel my work is wearing me down. I feel burnt out by my work.
9. I believe that with my work I am positively influencing the lives of my patients.
10. I have become more insensitive to people since I have practised this profession.
11. I am worried that this job is hardening me emotionally.
12. I feel very energetic in my work.
13. I feel frustrated in my work.
14. I think I work too much.
15. I don't really care what happens to some of my patients.
16. Working directly with people causes me stress.
17. I feel like I can easily create a pleasant atmosphere for my patients.
18. I feel motivated after working with my patients.
19. I think I achieve a lot of useful things in my profession.
20. I feel finished in my work, at the limit of my possibilities.
21. In my work I deal with problems emotionally very calmly.
22. I feel like patients blame me for some of their problems.

Table S2 — Connor-Davidson Resilience Scale version adapted to Spanish.

Likert-type answer with a score of 0-4.

0= Totally disagree

1= Disagree

2= Neither agree nor disagree

3= Agree

4= Totally agree

1. I adapt to changes.
2. I can handle any situation.
3. I see the positive side of things.
4. I can handle myself well despite pressure or stress
5. After a serious setback I usually `get back on track´.
6. I manage to achieve my goals despite the difficulties.
7. I can stay focused under pressure.
8. I hardly get discouraged by failures.
9. I define myself as a strong person.
10. I can handle unpleasant feelings.
